# Crystal structure of 3-(4-bromo­phenyl­sulfon­yl)-2,5,6-trimethyl-1-benzo­furan

**DOI:** 10.1107/S1600536814022119

**Published:** 2014-10-11

**Authors:** Hong Dae Choi, Uk Lee

**Affiliations:** a Department of Chemistry, Dongeui University, San 24 Kaya-dong, Busanjin-gu, Busan 614-714, Republic of Korea; b Department of Chemistry, Pukyong National University, 599-1 Daeyeon 3-dong, Nam-gu, Busan 608-737, Republic of Korea

**Keywords:** crystal structure, benzo­furan, 4-bromo­phen­yl, Br⋯π contacts and and C—H⋯π hydrogen bonds.

## Abstract

In the title compound, C_17_H_15_BrO_3_S, the dihedral angle between the planes of the benzo­furan ring system [r.m.s. deviation = 0.015 (2) Å] and the 4-bromo­phenyl ring is 89.29 (6)°. In the crystal, mol­ecules are linked into a chain along the *b*-axis direction by C—H⋯π hydrogen bonds and C—Br⋯π [3.626 (1) Å] inter­actions.

## Abstract 2

In 3-(4-bromo­phenyl­sulfon­yl)-2,5,6-trimethyl-1-benzo­furan, mol­ecules are linked into a chain along the *b*-axis direction by C—H⋯π hydrogen bonds and C—Br⋯π inter­actions.

## Chemical context   

Mol­ecules containing a benzo­furan ring show significant biological properties, such as anti­bacterial and anti­fungal (Aslam *et al.*, 2009 ▶), anti­tumor and anti­viral (Galal *et al.*, 2009 ▶) and anti­microbial activities (Wahab Khan *et al.*, 2005 ▶), and are potential inhibitors of β-amyloid aggregation (Ono *et al.*, 2002 ▶). Benzo­furan compounds occur widely in nature (Akgul & Anil, 2003 ▶; Soekamto *et al.*, 2003 ▶). As a part of our continuing project concerning 3-(aryl­sulfon­yl)-2,5,7-trimethyl-1-benzo­furan derivatives, we report herein on the synthesis and crystal structure of the title compound.
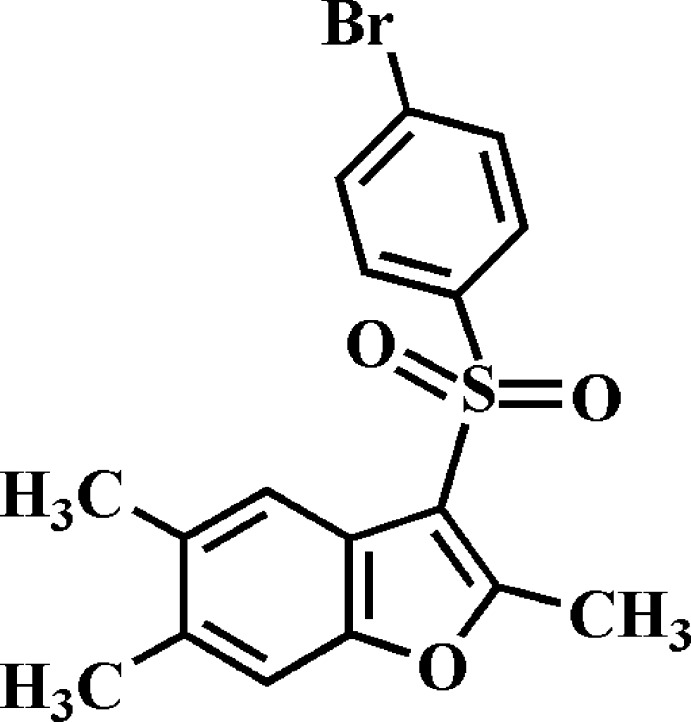


## Structural commentary   

**Figure 1:**
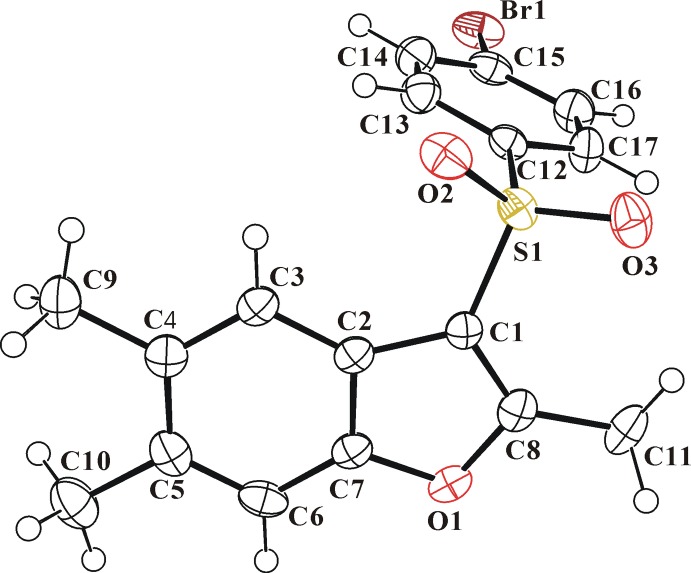
The mol­ecular structure of the title compound with the atom-numbering scheme. Displacement ellipsoids are drawn at the 50% probability level. H atoms are shown as small spheres of arbitrary radius.

In the title mol­ecule (Fig. 1 ▶), the benzo­furan unit (O1/C1–C8) is essentially planar, with a mean deviation of 0.015 (2) Å from the mean plane defined by the nine constituent atoms. The 4-bromo­phenyl ring (C12–C17) is inclined to the benzo­furan ring by 89.29 (6)°. The title compound crystallized in the non-centrosymmetric space group *Pc* in spite of having no asymmetric C atoms.

## Supra­molecular features   

**Figure 2:**
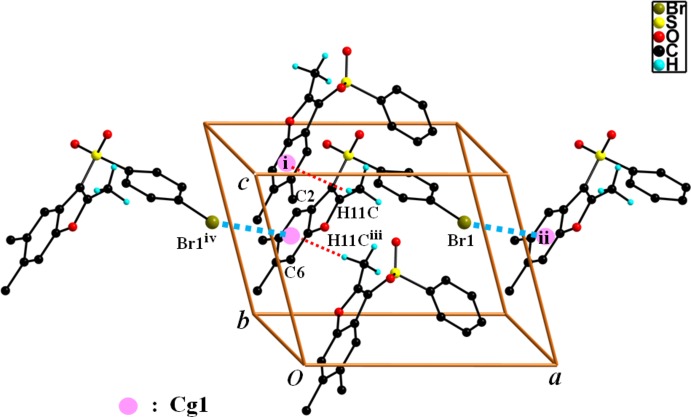
A view of the C—H⋯π and C—Br⋯π inter­actions (dotted lines) in the crystal structure of the title compound. H atoms not participating in hydrogen bonding have been omitted for clarity. [Symmetry codes: (i) *x*, −*y* + 1, *z* + 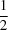; (ii) *x* + 1, *y*, *z*; (iii) *x*, −*y* + 1, *z* − 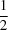; (iv) *x* − 1, *y*, *z*.]

In the crystal, mol­ecules are linked into a chain along the *b*-axis direction by C—H⋯π hydrogen bonds (Fig. 2 ▶ and Table 1 ▶), and by C15—Br1⋯π inter­actions between the Br atom and the benzene ring of a neighbouring mol­ecule with a Br1⋯*Cg*1^ii^ separation of 3.626 (1) Å [illustrated in Fig. 2 ▶; *Cg*1 is the centroid of the C2–C7 benzene ring; symmetry code: (ii) *x* + 1, *y*, *z*].

## Database survey   

A search of the Cambridge Structural Database (Version 5.35, last update May 2014; Groom & Allen, 2014 ▶) for 3-(aryl­sulfon­yl)benzo­furan gave 66 hits. Six of these are 3-aryl­sulfonyl-2,5,7-trimethyl-1-benzo­furan derivatives whose structures are closely related to that of the title compound. In these six compounds, the dihedral angle between the aryl­sulfonyl ring and the benzo­furan ring varies from *ca* 72.67° in 3-(4-fluoro­phenyl­sulfon­yl)-2,5,7-trimethyl-1-benzo­furan (Choi *et al.*, 2010 ▶) to 87.61° in 3-(2-fluoro­phenyl­sulfon­yl)-2,5,7-trimethyl-1-benzo­furan (Choi *et al.*, 2014 ▶). These dihedral angles are slightly smaller than the same angle of the title compound [89.29 (6)°].

## Synthesis and crystallization   

The starting material 3-(4-bromo­phenyl­sulfan­yl)-2,5,6-tri­methyl-1-benzo­furan was prepared by a literature method (Choi *et al.*, 1999 ▶). 3-Chloro­per­oxy­benzoic acid (77%, 448 mg, 2.0 mmol) was added in small portions to a stirred solution of 3-(4-bromo­phenyl­sulfan­yl)-2,5,6-trimethyl-1-benzo­furan (312 mg, 0.9 mmol) in di­chloro­methane (30 ml) at 273 K. After being stirred at room temperature for 10 h, the mixture was washed with saturated sodium bicarbonate solution (2 × 10 ml) and the organic layer was separated, dried over magnesium sulfate, filtered and concentrated at reduced pressure. The residue was purified by column chromatography (hexa­ne–ethyl acetate, 2:1 *v*/*v*) to afford the title compound as a colorless solid [yield 77%, 263 mg; m.p. 452–453 K; *R*_F_ = 0.58 (hexa­ne–ethyl acetate, 4:1 *v*/*v*)]. Single crystals suitable for X-ray diffraction were prepared by slow evaporation of a solution of the title compound (24 mg) in ethyl acetate (20 ml) at room temperature.

## Refinement   

Crystal data, data collection and structure refinement details are summarized in Table 2 ▶. All H atoms were positioned geometrically and refined using a riding model, with C—H = 0.95 for aryl and 0.98 Å for methyl H atoms, and with *U*_iso_(H) = 1.2*U*_eq_(C) for aryl and 1.5*U*_eq_(C) for methyl H atoms.

## Supplementary Material

Crystal structure: contains datablock(s) I. DOI: 10.1107/S1600536814022119/rn2128sup1.cif

Structure factors: contains datablock(s) I. DOI: 10.1107/S1600536814022119/rn2128Isup2.hkl

Supporting information file. DOI: 10.1107/S1600536814022119/rn2128Isup3.cml

CCDC reference: 1027923

Additional supporting information:  crystallographic information; 3D view; checkCIF report

## Figures and Tables

**Table 1:** Hydrogen-bond geometry (, ) *Cg*1 is the centroid of the C2C7 benzene ring.

*D*H*A*	*D*H	H*A*	*D* *A*	*D*H*A*
C11H11*B* *Cg*1^i^	0.98	2.89	3.504(3)	122

Symmetry code: (i) 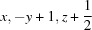.

**Table 2:** Experimental details

Crystal data	
Chemical formula	C_17_H_15_BrO_3_S
*M*_r_	379.26
Crystal system, space group	Monoclinic, *P* *c*
Temperature (K)	173
*a*, *b*, *c* ()	11.3395(3), 8.0093(2), 9.0439(2)
()	108.800(1)
*V* (^3^)	777.56(3)
*Z*	2
Radiation type	Mo *K*
(mm^1^)	2.79
Crystal size (mm)	0.21 0.17 0.15

Data collection	
Diffractometer	Bruker *SMART* APEXII CCD
Absorption correction	Multi-scan (*SADABS*; Bruker, 2009 ▶)
*T*_min_, *T*_max_	0.643, 0.746
No. of measured, independent and observed [*I* > 2(*I*)] reflections	12777, 3392, 3136
*R*_int_	0.030
(sin /)_max_ (^1^)	0.639

Refinement	
*R*[*F* ^2^ > 2(*F* ^2^)], *wR*(*F* ^2^), *S*	0.024, 0.056, 1.00
No. of reflections	3392
No. of parameters	202
No. of restraints	2
H-atom treatment	H-atom parameters constrained
_max_, _min_ (e ^3^)	0.32, 0.37
Absolute structure	Flack (1983 ▶), 1710 Friedel pairs
Absolute structure parameter	0.001(6)

Computer programs: *APEX2* and *SAINT* (Bruker, 2009 ▶), *SHELXS97* and *SHELXL97* (Sheldrick, 2008 ▶), *ORTEP-3 for Windows* (Farrugia, 2012 ▶) and *DIAMOND* (Brandenburg, 1998 ▶).

## supplementary crystallographic information

### Crystal data

**Table:**

C_17_H_15_BrO_3_S	*F*(000) = 384
*M**_r_* = 379.26	*D*_x_ = 1.620 Mg m^−^^3^
Monoclinic, *P**c*	Melting point = 453–452 K
Hall symbol: P -2yc	Mo *K*α radiation, λ = 0.71073 Å
*a* = 11.3395 (3) Å	Cell parameters from 6185 reflections
*b* = 8.0093 (2) Å	θ = 2.5–27.5°
*c* = 9.0439 (2) Å	µ = 2.79 mm^−^^1^
β = 108.800 (1)°	*T* = 173 K
*V* = 777.56 (3) Å^3^	Block, colourless
*Z* = 2	0.21 × 0.17 × 0.15 mm

### Data collection

**Table:**

Bruker SMART APEXII CCD diffractometer	3392 independent reflections
Radiation source: rotating anode	3136 reflections with *I* > 2σ(*I*)
Graphite multilayer monochromator	*R*_int_ = 0.030
Detector resolution: 10.0 pixels mm^-1^	θ_max_ = 27.0°, θ_min_ = 1.9°
φ and ω scans	*h* = −14→14
Absorption correction: multi-scan (*SADABS*; Bruker, 2009)	*k* = −10→10
*T*_min_ = 0.643, *T*_max_ = 0.746	*l* = −11→11
12777 measured reflections	

### Refinement

**Table:**

Refinement on *F*^2^	Secondary atom site location: difference Fourier map
Least-squares matrix: full	Hydrogen site location: difference Fourier map
*R*[*F*^2^ > 2σ(*F*^2^)] = 0.024	H-atom parameters constrained
*wR*(*F*^2^) = 0.056	*w* = 1/[σ^2^(*F*_o_^2^) + (0.0201*P*)^2^] where *P* = (*F*_o_^2^ + 2*F*_c_^2^)/3
*S* = 1.00	(Δ/σ)_max_ = 0.001
3392 reflections	Δρ_max_ = 0.32 e Å^−^^3^
202 parameters	Δρ_min_ = −0.37 e Å^−^^3^
2 restraints	Absolute structure: Flack (1983), 1710 Friedel pairs
Primary atom site location: structure-invariant direct methods	Absolute structure parameter: −0.001 (6)

### Special details

**Table:**

Experimental. ^1^H NMR (δ p.p.m., CDCl_3_, 400 Hz): 7.85 (d, J = 8.56 Hz, 2H), 7.63 (d, J = 8.21 Hz, 2H), 7.58 (s, 1H), 7.20(s, 1H), 2.76 (s, 3H), 2.34 (s, 3H), 2.32 (s, 3H)).
Geometry. All esds (except the esd in the dihedral angle between two l.s. planes) are estimated using the full covariance matrix. The cell esds are taken into account individually in the estimation of esds in distances, angles and torsion angles; correlations between esds in cell parameters are only used when they are defined by crystal symmetry. An approximate (isotropic) treatment of cell esds is used for estimating esds involving l.s. planes.
Refinement. Refinement of F^2^ against ALL reflections. The weighted R-factor wR and goodness of fit S are based on F^2^, conventional R-factors R are based on F, with F set to zero for negative F^2^. The threshold expression of F^2^ > 2sigma(F^2^) is used only for calculating R-factors(gt) etc. and is not relevant to the choice of reflections for refinement. R-factors based on F^2^ are statistically about twice as large as those based on F, and R- factors based on ALL data will be even larger.

### Fractional atomic coordinates and isotropic or equivalent isotropic displacement parameters (Å^2^)

**Table:**

	*x*	*y*	*z*	*U*_iso_*/*U*_eq_	
Br1	0.89314 (3)	0.76274 (4)	0.54426 (3)	0.04023 (8)	
S1	0.49513 (5)	0.71962 (7)	0.90695 (6)	0.02534 (13)	
O1	0.27996 (15)	0.39863 (18)	0.61667 (18)	0.0275 (4)	
O2	0.44621 (15)	0.8818 (2)	0.92194 (19)	0.0325 (4)	
O3	0.54542 (16)	0.6177 (2)	1.04338 (19)	0.0357 (4)	
C1	0.3808 (2)	0.6097 (2)	0.7663 (2)	0.0212 (4)	
C2	0.2883 (2)	0.6820 (3)	0.6333 (2)	0.0229 (4)	
C3	0.2506 (2)	0.8416 (3)	0.5766 (3)	0.0256 (5)	
H3	0.2894	0.9370	0.6343	0.031*	
C4	0.1567 (2)	0.8602 (3)	0.4364 (3)	0.0278 (5)	
C5	0.0994 (2)	0.7185 (3)	0.3471 (3)	0.0301 (6)	
C6	0.1362 (2)	0.5613 (3)	0.4028 (3)	0.0305 (5)	
H6	0.0986	0.4653	0.3451	0.037*	
C7	0.22934 (19)	0.5462 (3)	0.5449 (3)	0.0239 (5)	
C8	0.3733 (2)	0.4408 (3)	0.7508 (3)	0.0258 (5)	
C9	0.1161 (3)	1.0346 (3)	0.3779 (3)	0.0373 (6)	
H9A	0.1698	1.1164	0.4489	0.056*	
H9B	0.0295	1.0520	0.3740	0.056*	
H9C	0.1224	1.0485	0.2731	0.056*	
C10	−0.0008 (3)	0.7398 (3)	0.1912 (3)	0.0429 (7)	
H10A	−0.0211	0.6310	0.1397	0.064*	
H10B	0.0289	0.8152	0.1255	0.064*	
H10C	−0.0755	0.7872	0.2071	0.064*	
C11	0.4438 (3)	0.3001 (3)	0.8427 (3)	0.0360 (6)	
H11A	0.4959	0.3405	0.9453	0.054*	
H11B	0.4968	0.2505	0.7876	0.054*	
H11C	0.3856	0.2157	0.8564	0.054*	
C12	0.6123 (2)	0.7434 (3)	0.8190 (3)	0.0238 (5)	
C13	0.6082 (2)	0.8757 (3)	0.7190 (3)	0.0303 (5)	
H12	0.5469	0.9605	0.7046	0.036*	
C14	0.6935 (2)	0.8833 (3)	0.6407 (3)	0.0334 (6)	
H13	0.6915	0.9737	0.5719	0.040*	
C15	0.7818 (3)	0.7605 (3)	0.6615 (3)	0.0293 (5)	
C16	0.7893 (2)	0.6306 (3)	0.7645 (3)	0.0349 (6)	
H15	0.8527	0.5485	0.7810	0.042*	
C17	0.7032 (2)	0.6216 (3)	0.8436 (3)	0.0303 (5)	
H16	0.7065	0.5325	0.9141	0.036*	

### Atomic displacement parameters (Å^2^)

**Table:**

	*U*^11^	*U*^22^	*U*^33^	*U*^12^	*U*^13^	*U*^23^
Br1	0.03614 (13)	0.05357 (16)	0.03812 (15)	−0.01553 (15)	0.02190 (11)	−0.01138 (13)
S1	0.0241 (3)	0.0324 (3)	0.0203 (3)	0.0013 (3)	0.0082 (2)	−0.0013 (2)
O1	0.0323 (9)	0.0210 (8)	0.0336 (9)	−0.0030 (7)	0.0169 (8)	−0.0009 (6)
O2	0.0313 (9)	0.0347 (9)	0.0323 (9)	0.0021 (7)	0.0112 (7)	−0.0124 (7)
O3	0.0357 (9)	0.0502 (11)	0.0226 (8)	0.0051 (8)	0.0114 (7)	0.0069 (7)
C1	0.0213 (11)	0.0242 (10)	0.0208 (10)	0.0003 (9)	0.0103 (9)	0.0011 (8)
C2	0.0228 (11)	0.0243 (11)	0.0255 (12)	−0.0008 (9)	0.0133 (9)	0.0007 (8)
C3	0.0251 (11)	0.0244 (11)	0.0317 (12)	0.0005 (9)	0.0153 (9)	0.0010 (9)
C4	0.0250 (12)	0.0338 (13)	0.0299 (13)	0.0008 (9)	0.0161 (10)	0.0038 (9)
C5	0.0234 (13)	0.0464 (14)	0.0242 (13)	0.0020 (10)	0.0129 (11)	0.0049 (10)
C6	0.0252 (12)	0.0384 (13)	0.0309 (14)	−0.0081 (10)	0.0134 (11)	−0.0089 (10)
C7	0.0238 (11)	0.0229 (11)	0.0296 (13)	−0.0006 (9)	0.0148 (10)	−0.0005 (9)
C8	0.0270 (12)	0.0294 (11)	0.0273 (12)	0.0029 (9)	0.0174 (10)	0.0043 (9)
C9	0.0347 (13)	0.0419 (16)	0.0367 (13)	0.0083 (12)	0.0133 (11)	0.0108 (11)
C10	0.0337 (15)	0.0618 (19)	0.0312 (16)	0.0002 (12)	0.0075 (13)	0.0040 (12)
C11	0.0455 (15)	0.0251 (12)	0.0447 (16)	0.0075 (11)	0.0245 (13)	0.0107 (10)
C12	0.0223 (11)	0.0286 (11)	0.0198 (12)	−0.0025 (9)	0.0058 (10)	−0.0033 (8)
C13	0.0296 (12)	0.0283 (12)	0.0331 (13)	0.0018 (10)	0.0105 (10)	0.0006 (10)
C14	0.0346 (14)	0.0358 (14)	0.0305 (13)	−0.0040 (11)	0.0117 (11)	0.0044 (10)
C15	0.0241 (12)	0.0400 (13)	0.0256 (13)	−0.0092 (10)	0.0107 (10)	−0.0073 (9)
C16	0.0268 (12)	0.0400 (14)	0.0399 (14)	0.0035 (11)	0.0135 (11)	0.0043 (11)
C17	0.0280 (12)	0.0338 (13)	0.0308 (13)	0.0070 (10)	0.0115 (10)	0.0095 (10)

### Geometric parameters (Å, º)

**Table:**

Br1—C15	1.892 (3)	C9—H9A	0.9800
S1—O3	1.4354 (17)	C9—H9B	0.9800
S1—O2	1.4360 (17)	C9—H9C	0.9800
S1—C1	1.735 (2)	C10—H10A	0.9800
S1—C12	1.765 (3)	C10—H10B	0.9800
O1—C8	1.371 (3)	C10—H10C	0.9800
O1—C7	1.381 (2)	C11—H11A	0.9800
C1—C8	1.360 (3)	C11—H11B	0.9800
C1—C2	1.439 (3)	C11—H11C	0.9800
C2—C7	1.388 (3)	C12—C17	1.384 (3)
C2—C3	1.392 (3)	C12—C13	1.384 (3)
C3—C4	1.376 (3)	C13—C14	1.372 (3)
C3—H3	0.9500	C13—H12	0.9500
C4—C5	1.423 (3)	C14—C15	1.372 (4)
C4—C9	1.512 (3)	C14—H13	0.9500
C5—C6	1.370 (3)	C15—C16	1.381 (4)
C5—C10	1.508 (4)	C16—C17	1.386 (3)
C6—C7	1.381 (3)	C16—H15	0.9500
C6—H6	0.9500	C17—H16	0.9500
C8—C11	1.473 (3)		
			
O3—S1—O2	119.46 (10)	C4—C9—H9C	109.5
O3—S1—C1	109.74 (10)	H9A—C9—H9C	109.5
O2—S1—C1	107.95 (10)	H9B—C9—H9C	109.5
O3—S1—C12	107.50 (11)	C5—C10—H10A	109.5
O2—S1—C12	108.34 (11)	C5—C10—H10B	109.5
C1—S1—C12	102.55 (11)	H10A—C10—H10B	109.5
C8—O1—C7	106.92 (16)	C5—C10—H10C	109.5
C8—C1—C2	108.04 (19)	H10A—C10—H10C	109.5
C8—C1—S1	125.97 (18)	H10B—C10—H10C	109.5
C2—C1—S1	125.47 (16)	C8—C11—H11A	109.5
C7—C2—C3	118.3 (2)	C8—C11—H11B	109.5
C7—C2—C1	104.63 (19)	H11A—C11—H11B	109.5
C3—C2—C1	137.1 (2)	C8—C11—H11C	109.5
C4—C3—C2	119.6 (2)	H11A—C11—H11C	109.5
C4—C3—H3	120.2	H11B—C11—H11C	109.5
C2—C3—H3	120.2	C17—C12—C13	120.7 (2)
C3—C4—C5	120.8 (2)	C17—C12—S1	118.77 (18)
C3—C4—C9	118.7 (2)	C13—C12—S1	120.39 (19)
C5—C4—C9	120.4 (2)	C14—C13—C12	119.4 (2)
C6—C5—C4	119.7 (2)	C14—C13—H12	120.3
C6—C5—C10	119.8 (2)	C12—C13—H12	120.3
C4—C5—C10	120.6 (2)	C15—C14—C13	120.1 (2)
C5—C6—C7	118.3 (2)	C15—C14—H13	119.9
C5—C6—H6	120.8	C13—C14—H13	119.9
C7—C6—H6	120.8	C14—C15—C16	121.1 (3)
O1—C7—C6	126.2 (2)	C14—C15—Br1	120.3 (2)
O1—C7—C2	110.48 (19)	C16—C15—Br1	118.5 (2)
C6—C7—C2	123.3 (2)	C15—C16—C17	119.1 (2)
C1—C8—O1	109.9 (2)	C15—C16—H15	120.4
C1—C8—C11	134.3 (2)	C17—C16—H15	120.4
O1—C8—C11	115.8 (2)	C12—C17—C16	119.5 (2)
C4—C9—H9A	109.5	C12—C17—H16	120.2
C4—C9—H9B	109.5	C16—C17—H16	120.2
H9A—C9—H9B	109.5		
			
O3—S1—C1—C8	−26.6 (2)	C1—C2—C7—O1	0.4 (2)
O2—S1—C1—C8	−158.29 (18)	C3—C2—C7—C6	0.7 (3)
C12—S1—C1—C8	87.4 (2)	C1—C2—C7—C6	−177.6 (2)
O3—S1—C1—C2	162.75 (18)	C2—C1—C8—O1	−0.9 (2)
O2—S1—C1—C2	31.0 (2)	S1—C1—C8—O1	−172.93 (15)
C12—S1—C1—C2	−83.2 (2)	C2—C1—C8—C11	177.4 (3)
C8—C1—C2—C7	0.3 (2)	S1—C1—C8—C11	5.4 (4)
S1—C1—C2—C7	172.36 (16)	C7—O1—C8—C1	1.2 (2)
C8—C1—C2—C3	−177.5 (2)	C7—O1—C8—C11	−177.5 (2)
S1—C1—C2—C3	−5.4 (4)	O3—S1—C12—C17	25.8 (2)
C7—C2—C3—C4	0.3 (3)	O2—S1—C12—C17	156.21 (19)
C1—C2—C3—C4	177.8 (2)	C1—S1—C12—C17	−89.8 (2)
C2—C3—C4—C5	−1.3 (3)	O3—S1—C12—C13	−158.47 (19)
C2—C3—C4—C9	179.2 (2)	O2—S1—C12—C13	−28.1 (2)
C3—C4—C5—C6	1.4 (4)	C1—S1—C12—C13	85.9 (2)
C9—C4—C5—C6	−179.1 (2)	C17—C12—C13—C14	1.6 (4)
C3—C4—C5—C10	−178.2 (2)	S1—C12—C13—C14	−173.97 (18)
C9—C4—C5—C10	1.3 (4)	C12—C13—C14—C15	0.1 (4)
C4—C5—C6—C7	−0.4 (4)	C13—C14—C15—C16	−2.1 (4)
C10—C5—C6—C7	179.2 (2)	C13—C14—C15—Br1	176.07 (19)
C8—O1—C7—C6	177.0 (2)	C14—C15—C16—C17	2.3 (4)
C8—O1—C7—C2	−1.0 (2)	Br1—C15—C16—C17	−175.84 (18)
C5—C6—C7—O1	−178.3 (2)	C13—C12—C17—C16	−1.4 (4)
C5—C6—C7—C2	−0.6 (3)	S1—C12—C17—C16	174.32 (19)
C3—C2—C7—O1	178.70 (17)	C15—C16—C17—C12	−0.6 (4)

### Hydrogen-bond geometry (Å, º)

*Cg*1 is the centroid of the C2–C7 benzene ring.

**Table:**

*D*—H···*A*	*D*—H	H···*A*	*D*···*A*	*D*—H···*A*
C11—H11*B*···*Cg*1^i^	0.98	2.89	3.504 (3)	122

Symmetry code: (i) *x*, −*y*+1, *z*+1/2.
